# The US Anti-Prostitution Pledge: First Amendment Challenges and Public Health Priorities

**DOI:** 10.1371/journal.pmed.0040207

**Published:** 2007-07-24

**Authors:** Nicole Franck Masenior, Chris Beyrer

## Abstract

In order for an HIV organization to receive funding from the United States, grantees must explicitly oppose prostitution.

## Introduction

In January 2003, United States President George W. Bush announced the President's Emergency Plan for AIDS Relief (PEPFAR), the appropriation of $15 billion dollars for programs to combat the global HIV/AIDS epidemic, in his State of the Union address. Congress responded with the legislative authorization for the plan, the United States Leadership Against Global HIV/AIDS, Tuberculosis, and Malaria Act of 2003, and assigned seven primary implementing agencies, including the United States Agency for International Development (USAID) and the United States Department of Health and Human Services (HHS) [1]. Although the Act will expire in fiscal year 2008, the President has called on Congress once again to extend the plan for another five years and double the funding to $30 billion [2]. Within the detailed plan, Congress expressed concern about the social, cultural, and behavioral causes of HIV, specifically naming prostitution and sex trafficking as among the behavioral forces behind the spread of the virus. This legislation advanced a new policy goal for the US: the global eradication of prostitution [1].

Requirements for grantees were based on this explicit link between HIV prevention and the eradication of prostitution. In order to receive AIDS funds from the US, all grantees must have (1) a policy explicitly opposing prostitution and sex trafficking and (2) certification of compliance with the “Prohibition on the Promotion and Advocacy of the Legalization or Practice of Prostitution or Sex Trafficking,” which applies to all organization activities, including those with funding from private grants [1,3]. “The Prostitution Pledge,” as this requirement is often called, has evoked strong and mixed reactions. It has led some grantees, most prominently the government of Brazil, to reject US AIDS dollars altogether [4]. But it is the breadth of the requirement and its application to privately funded activities that has led to legal challenge of its constitutionality.

One ongoing lawsuit involves the Alliance for Open Society International (AOSI), the Open Society Institute, and Pathfinder International versus USAID, HHS, and the Centers for Disease Control and Prevention's Global AIDS Program. AOSI and the Open Society Institute filed a complaint against the US government on September 23, 2005 and moved for a preliminary injunction arguing that the pledge policy requirement cannot restrict activities supported by private funds [5]. Both cases were preceded by a similar case from DKT International, a nonprofit organization that provides family planning and HIV/AIDS prevention programs internationally [6].

The Brennan Center for Justice at NYU School of Law, the legal counsel representing AOSI, asked our Center for Public Health and Human Rights at Johns Hopkins to review the existing scientific evidence on strategies that effectively reduce rates of HIV among sex workers and to present our findings in a Declaration for the court (the full text of the Declaration is available at [7]). In this article, we present a brief summary of our findings and the main arguments used in AOSI's First Amendment case, followed by a discussion of how these data have created an ethical dilemma in policy decisions. Our methods are shown in Box 1.

**Box 1. Methods**

Our methods included a literature search using the electronic database PubMed, and a search for published, freely available reports on Web sites of HIV prevention organizations. All studies found on PubMed and used in the Declaration were published in peerreviewed journals. The following subject heading terms were used: “HIV/AIDS,” “HIV prevention,” “sexually transmitted diseases prevention,” “sex workers (male, female, and not gender speci. c),” “sex work,” “prostitutes,” “prostitution,” and “effective HIV strategies.” Bibliographies of articles were also reviewed, and only one unique report from the Joint United Nations Programme on HIV/AIDS was retrieved by this method.

Next, we reviewed published “Best Practices” reports from key global health organizations including the World Health Organization, the Joint United Nations Programme on HIV/AIDS, and the World Bank's HIV programs. In all the reports used, evidence-based studies shaped the guidelines and suggestions for future projects.

Lastly, we used a case studies approach to investigate HIV prevention policies and practices in those developing countries where there was evidence of control of HIV spread and relatively low and stable HIV prevalence among sex workers at national levels.

## Findings

One of our key findings was that the merging of the terms “prostitution” and “sex trafficking” in the Global AIDS Act is not accepted as standard language or practice by the scientific literature on HIV/AIDS or by international agencies with HIV prevention programs [8–10]. Trafficking in persons for any purpose is consistently seen as a criminal and human rights offense, and the subset of human trafficking related specifically to the sex industry is universally seen as among the most grievous of trafficking-related crimes [11]. While the law calls for opposing sex trafficking, we could find no entity that did not already oppose it. The same holds true for any form of prostitution involving children or minors—this was universally acknowledged as a crime and a human rights violation before the policy.

However, many organizations disagree with the Act's equation of all forms of prostitution with sex trafficking. The term prostitution itself is controversial—most groups working with persons who sell or trade sex for money use the terms “sex work” and “sex worker,” rather than “prostitute,” which is widely held to be stigmatizing and pejorative [8].

The core debate is that for many stakeholders, the category “sex workers” includes consenting adults who sell sex of their own volition, who are not trafficking victims, and who have called for recognition of their rights as workers, in settings that include Bangladesh, India, Thailand, Brazil, and the Dominican Republic [12–14]. A substantial body of peer-reviewed published studies suggests that the empowerment, organization, and unionization of sex workers can be an effective HIV prevention strategy and can reduce the other harms associated with sex work, including violence, police harassment, unwanted pregnancy, and the number of underage sex workers [13–16]. While sex work may be exploitative, and is illegal in many jurisdictions, sex worker advocates and HIV prevention program leaders generally concur that sex workers themselves need services, protection, peer outreach, and support from health professionals to reduce their risk of HIV infection [17–20]. While the language of the pledge does not mandate any specific changes in programs or services for sex workers, it does place funding restrictions on those programs with explicit policies calling for decriminalization or legalization of sex work.

## The First Amendment Case: HIV Prevention as “Protected Speech”

AOSI works in HIV/AIDS prevention activities primarily in Central Asia, where discrimination against sex workers is marked, governments are repressive, and police are often corrupt. AOSI uses “best practice” approaches, including harm reduction interventions with sex workers and intravenous drug users, to build local capacity and to move marginalized groups out of their disenfranchised status. Subsequent to filing suit, AOSI was warned by USAID, a major donor, that “advocating for the legalization of prostitution” or “organizing or unionizing prostitutes for the purpose of advocating for the legalization of prostitution” violates the policy requirement [5].

As plaintiff, AOSI argues that compelling the privately funded expression of a particular viewpoint in exchange for participation in a government-funded program is a violation of the First Amendment to the US Constitution, which assures freedom of expression [21]. The First Amendment protects the individual's right to speak, by free choice, either in harmony with the government's position or in direct opposition. If an individual or an organization expresses a different view than the government's, they cannot be punished by having certain opportunities withheld. The First Amendment provides constitutional rights to protect against this potential abuse of power.

For USAID, the Global AIDS Act does encourage and support organizations whose work with prostitutes includes “educat[ion]” and “counsel[ing]”, “help[ing] them escape,” and “provid[ing] them with condoms” [1]. However, one of Congress's and the Administration's many strategies for combating HIV, as detailed in the Act, is to eradicate prostitution [1]. Whether or not other approaches—such as unionizing sex workers to advocate for legal rights and protection—are more effective is an irrelevant argument in this case. The administration asserts that the government is allowed by the Constitution to choose a strategy that best serves their overall mission and to enforce a policy that ensures its mission will not be undermined by privately funded activities of the grantee. They argued that advocating for decriminalization, unionization, and tolerance of prostitution creates an environment of risky behavior and contradicts the program goals of the Global AIDS Act [21].

The government also suggests that the Spending Clause of the Constitution allows Congress to “attach conditions on the receipt of federal funds,” and that Congress “has repeatedly employed the power to further broad policy objectives by conditioning receipts of federal moneys upon compliance by the recipient with federal statutory and administrative directives.” The government therefore argues that organizations that apply for federal funds are free to not accept them if they don't agree with the requirements [21].

The Supreme Court has recognized this congressional power, but also its limits. Under the Unconstitutional Conditions Doctrine, Congress cannot ask recipients of their money to surrender vital constitutional rights [21]. Balancing the Court's Spending Clause, the First Amendment, and the potentially unconstitutional conditions of the Prostitution Pledge is the crux of this case.

## The Judge's Decision

In May 2006, the US District Court for the Southern District of New York issued a preliminary injunction against application of the policy requirement to AOSI and Pathfinder [21]. The court held that the Prostitution Pledge policy requirement unconstitutionally compelled speech, was viewpoint based, and placed an unconstitutional condition on the receipt of federal funds [21]. This was a ringing endorsement of the First Amendment. Almost four months following the Judge's decision, the government submitted an appeal to the US Court of Appeals for the Second Circuit. The Court heard this case on June 1, 2007. During the oral argument, the government's attorney stated that USAID and HHS are in the process of developing guidelines that would allow grantees to engage in the prohibited speech through an affiliate. A week later, USAID and HHS informed the court that the guidelines would be issued within 45 days, and that they would likely require the affiliate to be legally separate from the PEPFAR recipient, and to use largely separate physical premises and employees [22]. The Second Circuit has not yet indicated when and how it will rule.

The DKT case was appealed as well—to the US Court of Appeals for the D. C. Circuit. Its February 27, 2007 decision in favor of the government overturned the District Court's original ruling and held that the Prostitution Pledge requirement is constitutional [23]. The court based its holding on an assumption that USAID would allow PEPFAR recipients to engage in speech regarding prostitution, so long as they do it through an affiliate that receives no federal funds. A decision on whether DKT International will appeal the case is expected soon.

## Discussion

Reactions to the Global AIDS Act of 2003 and its prevention policies have varied, notably between groups already working on the ground and those applying for new funds. Brazil's National STD and AIDS Program made global headlines in May 2005 by refusing to adopt the Pledge and turning down some 40 million dollars from USAID for HIV prevention activities. Pedro Chequer, then Director of the National STD and AIDS Program, stated that “sex workers are part of implementing our AIDS policy and deciding how to promote it…They are our partners. How could we ask prostitutes to take a position against themselves?” [4].

How has the pledge requirement actually affected programs? While we found few published studies, several cases do suggest possible implications. In our view, the pledge has the potential to restrict programs for those it seeks to protect. Changes in policy have forced some non-US-based projects, such as the Médecins Sans Frontières–run Lotus Project in Svay Pak, Cambodia, to close [24]. Interviews with some 100 women in Svay Pak revealed that only a small number felt they had been forced into sex work, and a significant percentage sought to improve working conditions and safety. The Lotus Project began by offering a range of services to sex workers, from primary health care to English and computer lessons, while receiving funds from USAID for operations research. Within two years after the project's launch, Médecins Sans Frontières handed it over to a local organization, whose funding came primarily from USAID, in an effort to ensure sustainability. Around the same time, the Lotus Project had come on the radar of US activists working on human trafficking issues. After a number of raids on brothels in the area by US-funded anti-trafficking groups, sex workers experienced severely restricted mobility, resulting in limited access to health care and a reduced ability to earn a livelihood. The project's ability to respond effectively to the new situation was hindered by fear of being seen as promoting prostitution. Their freedom to deliver services based on best practices was limited. Eventually, funding from USAID diminished and the Lotus Project closed [24].

The experience of the Lotus Project contradicts a principal argument used in a friend of the court brief in support of USAID. This argument stated that “Organizations can oppose the prostitution industry without stigmatizing the individuals bought and sold in it…Helping victims while opposing the industry that exploits them is the best way to prevent HIV/AIDS, the best way to advance human rights, and the best way to fulfill the intent of the Act” [25]. We agree. Yet we would argue that the evidence suggests that as long as prostitution and sex trafficking remain conflated, women and men who voluntarily sell sex may be at risk of further marginalization and may, as witnessed by the Lotus Project, be less likely to receive the health, social, and education services they need to eventually move out of the industry.

In this polarized debate, neither side has been ready to surrender what it believes to be the best approach to HIV prevention among sex workers. Public health professionals have an obligation to demand that funding be driven by evidence-based strategies for addressing health and social problems, but evidence does not function in a political vacuum. The history of HIV prevention is all too full of programs that have proven to be politically unfeasible in the US despite overwhelming scientific evidence in their favor, such as the efficacy of needle and syringe exchange programs as HIV prevention tools for injecting drug users. The Federal Government continues its ban on federal funding for this intervention, despite endorsements from the Institute of Medicine, the US Surgeon General, and all of the major health professional associations [26].

These political realities have complicated responses to HIV prevention for sex work as well as for injection drug use. While sex work may be seen by some as inherently degrading, it is nevertheless the case that in many settings sex workers choose to continue to work and to demand preventive and other health services. The provision of support, goods, and services to sex workers who want and need them is a compelling ethical and public health priority. Building trust and showing care by providing sex workers with the tools necessary to stay alive, whether they be condoms, counseling, or a safe place to receive medical attention, is our duty as health professionals and as human beings. Whether these goals can be met if we must “oppose prostitution” is actively being argued in the courts, and perhaps more vitally, in the many settings where sex workers provide services societies continue to disdain and demand.

## Abbreviations

AOSI: Alliance for Open Society International
HHS: Department of Health and Human Services
PEPFAR: the President's Emergency Plan for AIDS Relief
USAID: United States Agency for International Development
